# Do isolates from pharyngeal and rectal swabs match blood culture bacterial pathogens in septic VLBW infants? A pilot, cross-sectional study

**DOI:** 10.1007/s00431-020-03788-0

**Published:** 2020-08-28

**Authors:** Letizia Capasso, Sergio Maddaluno, Clara Coppola, Pasquale Dolce, Giuseppe Schiano di Cola, Enrico Sierchio, Angela Carla Borrrelli, Maria Bagattini, Eliana Pia Esposito, Raffaele Zarrilli, Eleni Antonaki, Maria Rosaria Catania, Francesco Raimondi

**Affiliations:** 1 Department of Translational Medical Sciences - Division of Neonatology, University of Naples Federico II, Naples, Italy; 2 Department of Public Health, University of Naples Federico II, Naples, Italy; 3 NICU Monaldi Hospital, Naples, Italy; 4 Department of Molecular Medicine and Medical Biotechnology - Division of Bacteriology and Mycology, University of Naples Federico II, Naples, Italy

**Keywords:** Colonizing bacteria, Neonatal sepsis, VLBW infants, Blood culture, Superficial swab

## Abstract

Serial body site swabbing is used to monitor horizontal spread of aggressive bacterial species in the neonatal intensive care unit (NICU). Since colonization/carriage is thought to precede systemic infection, one might expect to retrieve colonizing pathogens from blood cultures. This hypothesis, however, has not been fully investigated in very low birth weight (VLBW) infants that are at high sepsis’ risk. The primary outcome was, in a population of VLBW infants with late-onset sepsis, the matching between blood culture results and pathogens isolated from rectal and nose/pharyngeal surveillance swabs in the preceding 2 weeks. The secondary outcomes were the site of swabbing and time interval from colonization to blood culture positivity. Out of 333 VLBW neonates, 80 (24%) were diagnosed with bacterial sepsis. In 46 (57%) neonates, the blood culture showed the same pathogen species cultured from a swab. Of these, 30 were isolated from infants with both body sites colonized with an average time interval of 3.5 days; 2/16 were isolated from rectal swabs and 14 /16 from nose/pharyngeal samples.

*Conclusion*: Our data show a fair correspondence between bacteria colonizing the nasopharynx and/or the rectum and pathogens later isolated from blood cultures. This association depends on the swabbing site, number of sites, and pathogen species. Although these data constitute valuable results, they are not sufficient for providing the sole base of a thoughtful clinical decision.

**Table:** 

**What is Known:** • *Body site’s colonization may precede systemic infection*. • *Little is known on this mechanism in VLBW infants that are at higher sepsis’ risk*. **What is New:** *•Colonizing bacteria partially correspond to pathogens of blood cultures in VLBW infants with sepsis*. • *Correspondence depends on swabbing site, number of sites, and pathogen species*.

**Electronic supplementary material:**

The online version of this article (10.1007/s00431-020-03788-0) contains supplementary material, which is available to authorized users.

## Introduction

Systemic infection is a common cause of neonatal mortality and morbidity as 25% of very low birth weight (VLBW) babies and up to 40% of extremely low birth weight (ELBW) infants experience at least a sepsis episode during NICU (neonatal intensive care unit) admission [1, 2].

Surveillance of healthcare-associated infections in III level NICU of University Hospital Federico II in Naples, Italy, during 2013–2017, showed that central line–associated blood stream infections were the most frequent infections (69.6%) occurring with a significant decreasing trend from the lowest to the highest birth weight classes [3].

Bacteria can follow different routes to invade the circulation. Biofilm-producing bacteria can proliferate on invasive vascular devices to disseminate and damage distant body districts [4]. Alternatively, pathogens eluding the barrier function offered by the skin or the mucosae [5] can translocate into the bloodstream triggering sepsis. Serial swabbing of selected body sites is a common strategy to monitor the horizontal pathogen spread in a NICU and prevent the occurrence of septic outbreaks [6–9]. Though the exact mechanism by which colonization progresses to infection is not fully understood, one might expect to retrieve in considerable proportion from blood cultures the same aggressive bacteria colonizing the patient. This association has not been extensively studied particularly in the fragile VLBW infant population that experienced the higher rate of sepsis [10, 11] and is the goal of the present work.

## Materials and methods

Our study population is all inborn VLBW (< 1500-g birthweight) infants admitted to our III level NICU between January 2015 and June 2019 who were diagnosed with late-onset sepsis according to the Vermont Oxford Network (VON) criteria, namely [12]:

- Culture-positive episodes occurring beyond 72 h of life
- For sepsis by coagulase-negative staphylococcus: pathogen recovered from either a central line or peripheral blood sample in association with one or more signs of generalized infection and treatment with 5 or more days of intravenous antibiotics after the above cultures were obtained
- For sepsis by other bacteria: bacterial pathogen recovered from blood culture
- For sepsis by fungi: fungus recovered from a blood culture obtained from either a central line or peripheral blood sample

- The clinical signs for the diagnosis of systemic infection recorded in the charts that led physicians to obtain a blood culture were apnea, mottled skin, temperature instability, feeding intolerance, significant abdominal distension, respiratory distress, and hemodynamic instability. Laboratory criteria indicative of sepsis were elevated CRP (cutoff = 1 mg/dl), elevated procalcitonin (cutoff > 0.5 ng/ml), abnormal leucocyte count (cutoff less than 5000/μl or more than 20,000/μl), and I/T ratio (cutoff > 0.2). Exclusion criteria were major congenital anomalies and documented TORCH group infections.

After our study population has been selected, with a cross-sectional design of the study, we retrospectively reviewed data recorded for each patients.

The primary outcome of the study was the number of septic episodes caused by the same microbial species colonizing the pharynx and/or the rectum in the previous 2 weeks over the total number of septic episodes.

The secondary outcomes are as follows:

- The detection rate for the individual microbial species and the site of swabbing (rectal or nose/pharyngeal); nose swabs were performed instead of pharyngeal swab when neonates were subjected to mechanical ventilation.
- The average time interval from colonization to blood culture positivity.

Additional data extracted for each patient included both general characteristics (mode of delivery; gender; Apgar at 5′ < 6; birth weight; gestational age; singleton vs twin gestation) and the common NICU outcomes (death; intraventricular hemorrhage (IVH) ≥ grade III; need for oxygen at 36 weeks post-menstrual age; necrotizing enterocolitis (NEC); duration of mechanical ventilation and/or noninvasive respiratory support (NIV); umbilical venous catheter (UVC) and central venous access (CVC) duration; length of hospital stay; patent ductus arteriosus (PDA) of hemodynamic significance as to the need for medical treatment with ibuprofen or surgical ligature).

### Microbiology

Blood cultures (1 ml minimum) were taken at admission and according to the attending physician order. All blood samples were placed in culture media for aerobes and anaerobes (BD BACTEC™, Becton Dickinson Company, Europe) and incubated at 36 °C ± 1 for 5 days. After incubation, subcultures were then plated on:

- MacConkey agar—incubated for 24 h at 36 °C
- Trypticase soy agar with 5% sheep blood—incubated for 24 h at 36 °C
- Columbia CNA (Colistin + Nalidixic Acid) agar with 5% sheep blood—incubated for 24 h at 36 °C
- Sabouraud dextrose agar with chloramphenicol and gentamicin—incubated for 48 h at 25 °C

Finally, bacterial identification and antimicrobial susceptibility testing were performed by the automatic Phoenix system (Becton Dickinson, Europe). The automatic Vitek2 system (bioMérieux Inc., France) was used to identify *Candida* species.

Results of the swab cultures and blood cultures are returned in the hospital intranet system and were recorded in the patient clinical chart.

According to our NICU infection surveillance protocol, all infants were swabbed once weekly in the nose/pharynx and the rectum. All samples were plated on:

- Agar MacConkey—incubated for 18–24 h at 37 °C
- Agar salt mannitol—incubated for 48 h at 37 °C

Pharyngeal samples were also plated on:

- Agar blood—incubated for 18–24 h at 37 °C
- Pseudomonas cetrimide agar—incubated for 48 h at 37 °C
- Agar Sabouraud chloramphenicol—incubated for 48 h at 25 °C

After incubation, bacterial identification was obtained with API (API-E, API-NE, API-Staf, API-Candida) manual identification solutions (bioMerieux Italia, Bagno a Ripoli, Italy).

At least three colonies were picked up from each sample from nose/pharynx or rectal swabs. All of them were analyzed for phenotypic identification and susceptibility to selected antimicrobials.

Microbial isolates from surveillance swabs were analyzed for the susceptibility to selected antimicrobials, which allow to identify them as sentinel pathogens, i.e., pathogens which most frequently are responsible for infections in healthcare facilities. In particular, monobactam and third-generation cephems were tested to assess ESBL activity in Enterobacteriaceae, piperacillin-tazobactam in *Pseudomonas*, meropenem to assess carbapenem resistance in Gram-negative bacteria, and oxacillin and vancomycin to assess resistance to methicillin and glycopeptides, respectively, in Gram-positive bacteria. All the selected antimicrobials tested on microbial isolates from surveillance swabs were included in the supplementary tables.

The local Ethical Committee approved the study. Meticulous care was exerted to ascertain that no major change had been made to the main NICU protocols that could have been relevant to the outcome during the study period.

### Statistical analysis

Data were presented as number of patients (%) for categorical variables and as mean (± standard deviation) for quantitative variables.

To test for significant differences among groups, ANOVA was used for quantitative variables and χ2 tests or Fisher’s exact test was used, as appropriate, for categorical variables.

McNemar test was used to verify whether the marginal proportion of positive to pharynx swab was equal or significantly different from the marginal proportion of positive to rectum swab. Cohen’s kappa was used as a measure of agreement between pharynx swab and rectum swab. Comparison between the sensitivity of swabs for the whole Gram-positive bacteria and the whole Gram-negative bacteria was performed using χ2 test with Yates’ continuity correction.

A post hoc power analysis was performed. Previous reports were focused on a wider age interval and/or a smaller number of pathogens [13–18]. Pooling their estimates, we found 0.33 was the average proportion of swabs matching a subsequent blood culture (null hypothesis). Given the width of our pathogens’ panel and the high sepsis risk in our VLBW infants, we hypothesized this proportion to be 0.5. Consequently, a sample size of 93 achieves 89% power to detect the 0.17 difference using a two-sided binomial test, with a significant level of 0.05.

This article is in agreement with the STROBE “Strengthening the Reporting of Observational Studies in Epidemiology” statement [19].

All statistical analyses were performed using the R software for statistical computing. A *p*-value < 0.05 was considered a statistical significance.

## Results

**Fig. 1:**
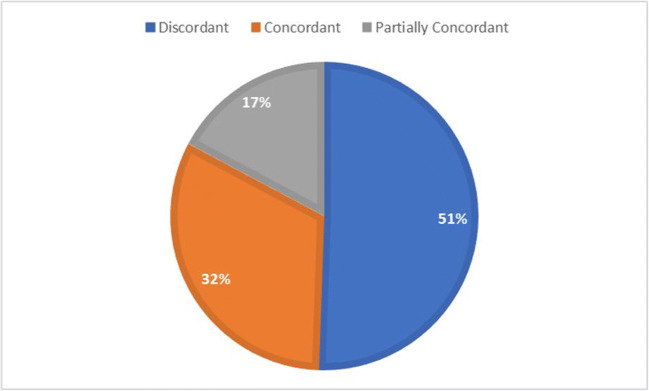
Distribution of correspondence between pathogens from blood cultures and microorganisms colonizing the nasopharynx and/or the rectum in the previous 2 weeks in the whole study population (*N* = 93). *Discordant*: neonates with blood pathogens undetected by the surveillance swabs. *Concordant*: positive nose/pharynx and rectum swabs correspondent with blood culture’s pathogen. *Partially concordant*: only nose/pharynx or rectal swab positive and concordant with blood culture’s pathogen

During the study period (January 2015–June 2019), 333 VLBW infants were surveilled in the NICU. Ninety-three (28%) infants were diagnosed with culture-proven sepsis. Most frequent pathogens responsible for sepsis were coagulase-negative staphylococci (*CONS*) (50; 53.7%), *Candida parapsilosis* (11; 11.8%), *Escherichia coli* (7; 7.5%), *Klebsiella pneumoniae* (6; 6.4%), *Staphylococcus aureus* (4; 4.3%), *Pseudomonas aeruginosa* (3; 3.2%), *Candida albicans* (2; 2.3%), and *Klebsiella oxytoca* (2; 2.3 %). Among these, 47 (50.5 %) patients had blood pathogens that went undetected by the surveillance swabs (Fig. 1), thirteen (28%) out 47 of which were yeasts belonging to *Candida* species (11 *C. parapsilosis* and 2 *C. albicans*). A flowchart showing the recruitment of patients is available online in the supplemental file.

Out of 80 registered bacterial sepsis episodes, forty-six (57 %) neonates had a blood pathogen matching the colonizing germs identified from surveillance swabs during the previous 2 weeks. A “phenotypic identification” was done considering the antibiogram profile of each blood culture and each swab culture and all the antibiogram profiles were identical between blood and swab cultures. We added the whole data as online supplement.

The characteristics of the cohort of the septic neonates and the correspondence between swabs and blood culture are reported in Table 1. Thirty (37.5%) out of 80 neonates had both swabs concordant with the pathogen isolated in the blood. Sixteen (20%) babies had only one swab matching the blood culture (14/16 pharynx; 2/16 rectum). Table 2 illustrates the relevant microbiology findings. Most frequent pathogens isolated from swabs and correspondent to blood cultures were:

- In nose/pharyngeal swabs: CONS (30/80; 37.5%), *K. pneumoniae* (5/80; 6.2%), E. *coli* (4/80; 5%), *S. aureus* (3/80; 3.7%), *P. aeruginosa* (1/80; 1.2%), and *K. oxytoca* (1/80; 1.2%)
- In rectal swabs: CONS (24/80; 30%), *K. pneumoniae* (5 /80; 6.2%), *E. coli* (2/80; 2.5%), and P. *aeruginosa* (1/80; 1.2%)

**Table 1:** Characteristics of neonates with proven sepsis

No. of neonates with proven sepsis: 93	Discordant *N* = 47 (50.5%)	Concordant *N* = 30 (32.2%)	Partially concordant *N* = 16 (17.2%)	*p*
Cesarean section	32 (72.7%)	16 (53.3%)	9 (60%)	0.219
Male Gender	23 (52.3%)	18 (60%)	6 (40%)	0.446
Apgar score at 5′ < 6 (no. of neonates)	24 (54.5%)	12 (40%)	6 (40%)	0.389
Birth weight (g)*	905 ± 296	927 ± 256	921 ± 327	0.950
Gestational age* (weeks)	27.7 ± 3.0	27.7 ± 3,0	27.5 ± 2.7	0,961
Singleton	34 (77%)	24 (80%)	12 (80%)	0.952
Mechanical ventilation (days)*	26.9 ± 40.4	11.1 ± 21.3	16.1 ± 18.7	0.107
NIV (days)*	16.1 ± 19.2	22.0 ± 22.5	17.7 ± 19.7	0.476
UVC (days)*	5.0 ± 2.2	6.0 ± 2.5	4.2 ± 2.7	**0.042**
CVC (days) *	34.7 ± 29.3	26.1 ± 19.0	25.3 ± 14.4	0.231
UVC + CVC (days)*	38.1 ± 31.1	21.7 ± 25.0	28.4 ± 17.48	**0.046**
IVH ≥ grade III	10 (22.7%)	5 (16.7%)	5 (33.4%)	0.450
O2 at 36 weeks (no. of neonates)	16 (36.4%)	9 (30%)	6 (40%)	0.637
NEC	1 (2.3%)	2 (6.7%)	0	0.430
Death (no. of neonates)	12 (27.3%)	3 (10%)	2 (13.3%)	0.147
Days of hospitalization*	75.5 ± 54.0	76.0 ± 38.4	69.4 ± 56.2	0.903
Ibuprofen for PDA (n° of neonates)	11 (25%)	6 (20%)	7 (46.7%)	0.151
PDA ligation (no. of neonates)	1 (2.3%)	0	1 (6.7%)	0.421

*Discordant*: neonates with blood pathogens undetected by the surveillance swabs. *Concordant*: positive nose/pharynx and rectum swabs correspondent with blood culture’s pathogen. *Partially concordant*: only nose/pharynx or rectal swab positive and concordant with blood culture’s pathogen

boldface indicates statistically significant results

*mean ± SD

*g* grams, *NIV* noninvasive respiratory support, *UVC* umbilical venous catheter, *CVC* central venous access, *IVH* intraventricular hemorrhage, *O*_*2*_ oxygen, *NEC* necrotizing enterocolitis, *PDA* patent ductus arteriosus

**Table 2:** Bacteria from blood cultures matching the colonizing germs identified from surveillance swabs during the previous 2 weeks

Bacteria detected in blood cultures	*CONS*	*E. coli*	*K. pneumoniae*	*K. oxytoca*	*P. aeruginosa*	*S. aureus*	Others#	Total
Concordant*	23	2	5	-	-	-	-	30
Nose/Pharynx*	7	2	-	1	1	3	**-**	14
Rectum*	1	-	-	-	1	-	**-**	2
Total swabs matching with blood cultures	31	4	5	1	2	3	-	46
Total blood cultures	50	7	6	2	3	4	8	80
Rate of detection	62%	57%	83%	50%	67%	75%	-	57%

Total neonates with one or two positive swabs matching with blood cultures = 46 (57%) on 80 neonates with bacterial sepsis

*Concordant*: positive nose/pharynx and rectal swabs matching blood cultures. Nose/*pharynx*: only nose/pharynx swab positive swabs matching blood cultures. *Rectum*: only rectal swab positive swabs matching blood cultures

*Pharyngeal swabs had a statistically significant higher concordance with blood cultures than rectal ones in all neonates with bacterial sepsis (McNemar test: *p* = 0.006. Cohen’s kappa = 0.61, which is a fair to good strength of agreement)

^#^*Others G +*: 4 *Enterococcus faecium*, 1 *Corynebacterium amycolatum*

*Others G −*: 2 *Serratia marcescens*, 1 *Enterobacter aerogenes*

The above isolated colonizing bacteria were tested for multidrug resistance; 3 *S. aureus* resulted as MRSA, 2 *K. pneumoniae* resulted as ESBL-positive and susceptible to carbapenems.

Sensitivity of rectal and nose/pharyngeal swabs for Gram-positive bacteria was compared with sensitivity of swabs for Gram-negative bacteria. For each group, we considered as correspondent to blood culture a positive rectal and/or pharyngeal swab (concordant and partially concordant swabs). The sensitivity resulted 58% for Gram-positive and 61% for Gram-negative and the difference was not statistically significant (*p* = 0.999).

The percentage of positive blood culture concordant with pharynx swab was equal to 0.55, while the percentage of concordant with rectum swab was 0.4. McNemar test suggested a statistically significant difference between these two marginal proportions (*p* = 0.006). Cohen’s kappa was equal to 0.61, which represents a fair to good strength of agreement [20] between the two swabs. The hypothesis that the agreement is the same as chance agreement (Cohen’s kappa = 0) was also rejected (*p* < 0.001).

Moreover, we computed the sensitivity of rectal and nose/pharyngeal swabs separately. Sensitivity of rectal swab for Gram-positive and Gram-negative was equal to 40 and 43%, respectively, and the difference was not statistically different (*p* = 0.995). Sensitivity of nose/pharyngeal swab for Gram-positive and Gram-negative was equal to 56 and 57%, respectively, and the difference was not statistically different (*p* = 0.999).

The average time interval from colonization to blood culture positivity was 8.5 days for rectal swabs, 4.5 days for pharyngeal samples, and 3.5 days when both swabs matched the blood pathogen.

Table 2 also reports the detection rate of at least one (pharynx or rectal) or both swabs for sepsis pathogen in the subsequent 2 weeks. *K. pneumoniae*-positive swab showed the highest rate of detection (83%), followed by *S. aureus* (75%). The overall rate of detection for Gram-negative bacterial sepsis was 57% (12/21). The overall rate of detection for Gram-positive bacterial sepsis was 57.6 % (34/59).

## Discussion

Our pilot study shows that bacterial pathogens isolated from blood cultures in septic VLBW babies have a fair chance (57%) of matching the bacteria found in our surveillance swabs in the previous 2 weeks. This association is species dependent with higher detection rates for *K. pneumoniae* and *P. aeruginosa* among Gram-negatives.

This potentially useful piece of clinical information is kept with previous findings. In a systematic review and meta-analysis, Seidel et al. [21] addressed the prognostic value of routine body surface screening for Gram-negative bacteria in a population of infants up to 12 months admitted to the NICU. Although the study population was very heterogeneous and the global sensitivity was only 41%, the pooled figure for *K. pneumoniae* was over twofold higher (92%). Among Gram-positive bacteria, our data found a high detection rate for *S. aureus* and CONS. In a series of preterm neonates affected by CONS sepsis, Soeorg H et al. [14] showed a genotypic similarity between blood isolates and those found in at least one out of two of rectal swabs performed on 72% of the patients in the previous week. Our lower detection rate may be explained by the single rectal swab taken in the time interval. At the same time, this suggests that increasing the frequency of the surveillance swabs might improve its sepsis prediction power.

Apart from CONS, we acknowledge to have a small number of individual species to perform an agreement analysis by type of bacteria. However, we computed the sensitivity of rectal and nose/pharyngeal swabs distinguishing between the whole Gram-positive bacteria and the whole Gram-negative bacteria retrieved in the subsequent blood cultures. The sensitivity values were only moderate and there was not a statistically significant difference for the two bacterial families.

Clinicians should bear in mind that compared with single swab, having both swabs (pharyngeal and rectal) positive for a pathogen increases the detection rate in the blood culture in a shorter time. Also, though figures are small, in our series, pharyngeal swabs had a statistically significant higher concordance with blood cultures than rectal ones considering all neonates suffering from bacterial sepsis. This result holds also if we perform the analysis separately for Gram-positive and Gram-negative families of bacteria.

Focusing on sepsis, we observed an increase of sepsis caused by *C. parapsilosis* during 2015 in the NICU [3] that had no match in the surveillance swabs. This data might have several explanations. First, we used agar Sabouraud chloramphenicol medium for pharyngeal samples only; this medium has been reported to detect 86.7% of the yeast colonies with a species specificity. Its detection rate is high for *C. albicans* but rather poor for *C. parapsilosis* [22]. The latter is mostly associated with central line–related bloodstream infections. Finally, during the study period, all our VLBW infants received a prophylactic regimen with fluconazole, which has high activity against *C. albicans* but much less against *C. parapsilosis* [23].

We acknowledge some obvious limitations to this study starting with the small number of patients. We believe that bigger figures and possibly a stricter surveillance protocol would have yielded stronger conclusions. In addition, no identification at species level was performed on CONS isolates from surveillance swabs, no molecular typing of the isolates was carried out both in surveillance swabs and blood culture, and the matching was performed on phenotypic criteria only. Furthermore, regarding the phenotypic identification of pathogens, we acknowledge that the lack of a complete antibiogram on microbial isolates from surveillance swabs, not only limited to its use in discovering sentinel pathogens, is a limit in determining a full relatedness of isolates.

## Conclusions

Our preliminary data show a moderate grade of matching between pathogens isolated from blood culture of septic VLBW infants and bacterial species from pharyngeal and/or rectal specimens in the previous 2 weeks. This association depends on the swabbing site, the number of sites, and the pathogen species. This pilot investigation provides useful clinical indications, although it is insufficient to constitute per se the base of a clinical decision. It also opens the way to larger, multicenter studies that are warranted to strengthen the correlation between bacterial colonizing body sites and pathogens causing a threatening disease such as neonatal sepsis.

## Electronic supplementary material

ESM 1 (DOCX 29 kb)

ESM 2 (DOCX 18 kb)

## Abbreviations

CONS: Coagulase-negative staphylococci
CVC: Central venous access
IVH: Intraventricular hemorrhage
NEC: Necrotizing enterocolitis
NIV: Noninvasive respiratory support
PDA: Patent ductus arteriosus
UVC: Umbilical venous catheter
VON: Vermont Oxford Network
