# Supplementary material for: Do isolates from pharyngeal and rectal swabs match blood culture bacterial pathogens in septic VLBW infants? A pilot, cross-sectional study
Source: Eur J Pediatr. 2020 Aug 28;180(3):799–806. doi: 10.1007/s00431-020-03788-0 (PMC7886719; doi:10.1007/s00431-020-03788-0)
Supplement: Supplementary file 1 — (DOCX 29 kb) [file 431_2020_3788_MOESM1_ESM.docx]

**Flow chart showing recruitment of patients**

Identification of surveillance swabs in the previous 2 weeks

Diagnosis of blood culture

proven late onset sepsis

N=93

All VLBW infants surveilled between January 2015-June 2019

N=333

Bacterial blood cultures matching surveillance swabs

N=46

Diagnosis of bacterial

proven late onset sepsis

N=80
