# Supplementary material for: Do isolates from pharyngeal and rectal swabs match blood culture bacterial pathogens in septic VLBW infants? A pilot, cross-sectional study
Source: Eur J Pediatr. 2020 Aug 28;180(3):799–806. doi: 10.1007/s00431-020-03788-0 (PMC7886719; doi:10.1007/s00431-020-03788-0)
Supplement: Supplementary file 2 — (DOCX 18 kb) [file 431_2020_3788_MOESM2_ESM.docx]

| **Patient** | Microbial isolate | **Blood cultures Antibiotype** | | | | **Swab Antibiotype** | | | |
| --- | --- | --- | --- | --- | --- | --- | --- | --- | --- |
|  |  | **AMO/CLAV** | **CFTX** | **CFTZ** | **MRP** | **AMO/CLAV** | **CFTX** | **CFTZ** | **MRP** |
| CS | *E. coli* | S | S | S | S | S | S | S | S |
| GM | *E. coli* | R | S | S | S | R | S | S | S |
| RM | *E. coli* | S | S | S | S | S | S | S | S |
| OV | *E. coli* | R | R | R | S | R | R | R | S |
| CC | *K. pneumoniae* | R | R | R | S | R | R | R | S |
| PGP | *K. pneumoniae* | R | R | R | S | R | R | R | S |
| VV | *K. pneumoniae* | S | S | S | S | S | S | S | S |
| KD | *K. pneumoniae* | S | S | S | S | S | S | S | S |
| BL | *K. pneumoniae* | S | S | S | S | S | S | S | S |
| AC | *K. oxytoca* | S | S | S | S | S | S | S | S |
| **Patient** | Microbial isolate | **Emocolture Antibiotype** | | | | **Swab Antibiotype** | | | |
|  |  | **PIP/TAZ** | | **MRP** | | **PIP/TAZ** | | **MRP** | |
| CA | *P. aeruginosa* | S | | S | | S | | S | |
| CD | *P. aeruginosa* | S | | S | | S | | S | |

*E. coli*: *Escherichia coli*; *K. pneumoniae*: *Klebsiella pneumoniae*; *K. oxytoca*: *Klebsiella oxytoca*; *E. cloacae*: *Enterobacter cloacae*; *P. aeruginosa*: *Pseudomonas aeruginosa*.

AMO/CLAV: Amoxicillin/clavulanic acid

CFTX: Cefotaxime

CFTZ: Cefotazidime

MRP: Meropenem

PIP/TAZ: Piperacillin/tazobactam

R: Resistant; S: susceptible

TOT: 4 *E. coli*, 5 *K. pneumoniae*, 1 *K. oxytoca*, 2 *P. aeruginosa*.

| **Patient** | **Microbial isolate** | **Blood cultures Antibiotype** | |  | **Swab Antibiotype** | |  |
| --- | --- | --- | --- | --- | --- | --- | --- |
|  |  | **OXA** | **VANCO** |  | **OXA** | **VANCO** |  |
| JJK | *S. aureus* | S | S |  | S | S |  |
| DV | *S. aureus* | R | S |  | R | S |  |
| TF | *S. aureus* | S | S |  | S | S |  |
| BF | CONS | R | S |  | R | S |  |
| CM | CONS | R | S |  | R | S |  |
| RE | CONS | R | S |  | R | S |  |
| KN | CONS | R | S |  | R | S |  |
| AGM | CONS | R | S |  | R | S |  |
| IP | CONS | R | S |  | R | S |  |
| PE | CONS | R | S |  | R | S |  |
| LRF | CONS | R | S |  | R | S |  |
| TR | CONS | R | S |  | R | S |  |
| CA | CONS | R | S |  | R | S |  |
| SS | CONS | R | S |  | R | S |  |
| RR | CONS | R | S |  | R | S |  |
| CMG | CONS | R | S |  | R | S |  |
| CC | CONS | R | S |  | R | S |  |
| LG | CONS | R | S |  | R | S |  |
| BA | CONS | R | S |  | R | S |  |
| BS | CONS | R | S |  | R | S |  |
| PS | CONS | R | S |  | R | S |  |
| NA | CONS | R | S |  | R | S |  |
| LG | CONS | R | S |  | R | S |  |
| GGP | CONS | R | S |  | R | S |  |
| DSE | CONS | R | S |  | R | S |  |
| MMF | CONS | R | S |  | R | S |  |
| DPD | CONS | R | S |  | R | S |  |
| RL | CONS | R | S |  | R | S |  |
| BG | CONS | R | S |  | R | S |  |
| SVM | CONS | R | S |  | R | S |  |
| GC | CONS | R | S |  | R | S |  |
| AA | CONS | R | S |  | R | S |  |
| DRG | CONS | R | S |  | R | S |  |
| MSM | CONS | R | S |  | R | S |  |

S. aureus: Staphylococcus aureus; CONS: coagulase-negative staphylococcus.

OXA: Oxacillin

VANCO: Vancomycin

R: Resistant; S: susceptible

TOT 31 CONS, 3 S. aureus.
